# Sec22b-dependent antigen cross-presentation is a significant contributor of T cell priming during infection with the parasite *Trypanosoma cruzi*

**DOI:** 10.3389/fcell.2023.1138571

**Published:** 2023-03-01

**Authors:** Lucía Biscari, Ma Carmen Maza, Cecilia Farré, Cintia Daniela Kaufman, Sebastian Amigorena, Manuel Fresno, Núria Gironès, Andrés Alloatti

**Affiliations:** ^1^ Instituto de Inmunología Clínica y Experimental de Rosario (IDICER), CONICET, Universidad Nacional de Rosario, Rosario, Argentina; ^2^ Departamento de Biología Molecular, Universidad Autónoma de Madrid (UAM), Madrid, Spain; ^3^ Centro de Biología Molecular Severo Ochoa (CSIC-UAM), Madrid, Spain; ^4^ Centro de Investigación y Producción de Reactivos Biológicos, Facultad de Ciencias Médicas, Universidad Nacional de Rosario, Rosario, Argentina; ^5^ Institut Curie, INSERM U932, Immunity and Cancer, PSL University, Paris, France; ^6^ Instituto de Investigación Sanitaria del Hospital Universitario de la Princesa, Madrid, Spain

**Keywords:** Sec22b SNARE protein, antigen cross-presentation, Trypanosoma cruzi, dendritic cells, CD8 + T cells, cre-loxp transgenic mice

## Abstract

Antigen cross-presentation is a vital mechanism of dendritic cells and other antigen presenting cells to orchestrate the priming of cytotoxic responses towards killing of infected or cancer cells. In this process, exogenous antigens are internalized by dendritic cells, processed, loaded onto MHC class I molecules and presented to CD8^+^ T cells to activate them. Sec22b is an ER-Golgi Intermediate Compartment resident SNARE protein that, in partnership with sintaxin4, coordinates the recruitment of the transporter associated with antigen processing protein and the peptide loading complex to phagosomes, where antigenic peptides that have been proteolyzed in the cytosol are loaded in MHC class I molecules and transported to the cell membrane. The silencing of Sec22b in dendritic cells primary cultures and conditionally in dendritic cells of C57BL/6 mice, critically impairs antigen cross-presentation, but neither affects other antigen presentation routes nor cytokine production and secretion. Mice with Sec22b conditionally silenced in dendritic cells (Sec22b^−/−^) show deficient priming of CD8^+^ T lymphocytes, fail to control tumor growth, and are resistant to anti-checkpoint immunotherapy. In this work, we show that Sec22b^−/−^ mice elicit a deficient specific CD8^+^ T cell response when challenged with sublethal doses of *Trypanosoma cruzi* trypomastigotes that is associated with increased blood parasitemia and diminished survival.

## 1 Introduction

Dendritic cells (DCs) are the most competent cells for priming cytotoxic CD8^+^ T lymphocytes, as they can efficiently present antigenic peptides associated with major histocompatibility complex class I (MHC class I) molecules together with other signals, such as cytokines and costimulatory molecules expressed in response to the environment and the antigenic source (Jung et al., 2002; Reis E Sousa, 2006; Zammit and Lefrançois, 2006). Most cells, including DCs, can present intracellular antigens loaded onto MHC class I molecules through the endogenous MHC class I antigen presentation pathway, also known as direct presentation (DPt). These intracellular antigens are processed in the cytosol by the proteasome, transported by the transporter associated with antigen processing (TAP) to the endoplasmic reticulum (ER) and trimmed by endopeptidases. The resulting peptides are loaded onto MHC class I molecules and recruited to the cell membrane. However, DCs can also internalize foreign antigens from the extracellular milieu, process them and present them bound to MHC class I molecules to stimulate naïve CD8^+^ T cell responses, a process known as cross-presentation (XPt) (Joffre et al., 2012; Blum et al., 2013; Kotsias et al., 2019). DCs are particularly efficient antigen presenting cells, as they show unique characteristics -such as delayed phago-lysosomal fusion and early recruitment of the NOX2 to phagosomes-that promote a mild degradative niche for antigens, hence favoring antigen processing and presentation instead of pathogen elimination (Savina and Amigorena, 2007; Alloatti et al., 2015; 2016; Embgenbroich and Burgdorf, 2018).

Within the different subpopulations of DCs, conventional type 1 DCs (cDC1) have been characterized as efficient cross-presenting cells *in vivo* in both humans and mice (Den Haan et al., 2000; Bedoui et al., 2009; Balan et al., 2019). In these cells, two main XPt routes have been described: vacuolar and cytosolic, according to the compartment where antigen processing occurs. However, the cytosolic pathway has been studied more and has collected more experimental evidence (Cruz et al., 2017). In the cytosolic pathway, internalized antigens are partially degraded in the phagosome or endosome and are then translocated to the cytosol where they are ubiquitinated and further processed by the proteasome. The peptides obtained are transported by TAP to either the ER or back to the phagosome to be loaded onto MHC class I molecules (Ackerman et al., 2003; Joffre et al., 2012; Wagner et al., 2012; Nair-Gupta and Blander, 2013; Cruz et al., 2017; Kotsias et al., 2019). Peptide loading in phagosomes require both TAP and the peptide-loading complex (the rest of the machinery involved in transporting and loading the peptides onto MHC class I molecules) to be recruited from the ER to the endocytic or phagocytic compartments, and in this process the SNARE protein Sec22b plays an essential role. Sec22b is a resident protein of the ER-Golgi intermediate compartment, and throughout the interaction with the SNARE protein syntaxin 4 –localized in phagosomes–promotes the fusion between these two compartments and thus the recruitment of ER cargo to phagosomes. (Cebrian et al., 2011).

A few animal models have been developed to study the relevance of XPt in the priming of immune responses *in vivo*. One of them is the Batf3^−/−^ mouse line, which lacks the cDC1 subset (Hildner et al., 2008). These mice show impaired antigen XPt but also a decreased capacity to secrete IL-12 and other important pro-inflammatory cytokines. Recently, a mouse model with WDFY4 conditionally silenced in cDC1s showed deficient XPt but a normal cytokine profile (Theisen et al., 2018). Our group has developed and characterized another mouse line with Sec22b conditionally excised in DC populations, which presents a clear phenotype of impaired antigen XPt but functional DPt and classic presentation in MHC class II molecules (Alloatti et al., 2017). These mice (from now on Sec22b^−/−^) fail to cross-prime CD8^+^ T cells to initiate responses against dead cells and tumor antigens if compared to their littermates (from now on Sec22b^+/+^) (Alloatti et al., 2017), but to our knowledge they were not studied in the context of infectious diseases that require cytotoxic responses to control the pathogen replication. For this reason, in this work we set out to further characterize this line of mice in a context of infection with the intracellular parasite *Trypanosoma cruzi*, causative agent of Chagas’ disease in human.

In mammals, *T. cruzi* trypomastigotes infect host cells establishing a parasitophorous vacuole that is subsequently lysed, releasing the newly formed amastigotes to the cytosol of the infected cell, where they replicate. Like in many intracellular infections, CD8^+^ T cells are essential effectors of the immune response against *T. cruzi*, as demonstrated by experimental evidence (Tarleton, 1990; Tarleton et al., 1992; 1996; Rottenberg et al., 1993). This CD8^+^ T cell response is robust and is critical for the control of the parasite load, although it develops more slowly when compared to the response against other infections (Tarleton et al., 1994; Tzelepis et al., 2006). Additionally, during *T. cruzi* infection, the cytotoxic response is biased to certain immunodominant epitopes (e.g., TsKb20 in C57BL/6 mice) derived from the *T. cruzi* family of trans-sialidase proteins (Rosenberg et al., 2010; Tarleton, 2015; Ferragut et al., 2021). Although the CD8^+^ T cell response has been extensively studied, the contribution of the different mechanisms of antigen presentation (DPt or XPt) to elicit the response during parasitic infection has not been addressed. Besides, antigen XPt is important in the development of the immune response against infection with other related parasites such as *Toxoplasma gondii* and *Leishmania major*, and Batf3^−/−^ mice are extremely susceptible to infection with these parasites (Bertholet et al., 2006; Mashayekhi et al., 2011; Ashok et al., 2014; Martínez-López et al., 2015). We thus aimed to characterize the Sec22b^−/−^ mouse line in the context of the infection with *T. cruzi,* and thereby to contribute to the study of the role of XPt during this parasitic infection. Analyzing spleens and lymph nodes from Sec22b^−/−^ mice infected with *T. cruzi*, we found that XPt is an important mechanism in the priming of *T. cruzi*-specific CD8^+^ T cells. Furthermore, Sec22b^*−/−*^ mice showed increased parasitemia than Sec22b^+/+^ (littermate controls), and lost weight until they finally died, while 100% of Sec22b^+/+^ mice survived at the time analyzed.

## 2 Materials and methods

### 2.1 Experimental animals

Mice were maintained with a supply of food and sterile water *ad libitum*, exposed to a 12-h light/dark cycle in specific pathogen-free conditions in the animal facility. Sec22b^−/−^ and Sec22b^+/+^ male mice were provided by the Curie Institute. All animals were 8–20 weeks old. As per mice production, Sec22b^−/−^ mice together with their Sec22b^+/+^ littermates were generated by crossing Sec22b^flox/WT^ mice with CD11c^Cre^ mice. 25% of the progeny have the Sec22b^flox/WT Cre+^ genotype. By means of another mating between mice obtained in the first cross, Sec22b^flox/flox Cre+^ (Sec22b^−/−^) mice and Sec22b^WT/WT Cre+^ (Sec22b^+/+^) mice were generated. Sec22b^−/−^ mice express the Cre recombinase under the CD11c promoter (specific to DCs) which catalyzes the ablation of an essential exon - flanked by LoxP cleavage sites of the Cre recombinase, in Sec22b^flox/flox^ mice - of the Sec22b protein. Therefore, Sec22b^−/−^ mice present a non-functional Sec22b protein only in DCs (Alloatti et al., 2017). As a method of euthanasia, a CO_2_ chamber was used followed by cervical dislocation. All procedures were approved and performed following the guidelines and recommendations of the animal ethical committee (IUCAC) of the School of Medical Sciences of the National University of Rosario (res. 6157/2018).

### 2.2 Parasites and infection

Sec22b^−/−^ and Sec2b^+/+^ mice were intraperitoneally (IP) infected with 2,000 blood trypomastigotes of *T. cruzi* Y strain. Previously, the parasites were thawed, and inoculated into IFNγ receptor KO C57BL/6 recipient mice as detailed in Sanoja et al., 2013; Castro et al., 2007. After this initial passage, blood was collected and trypomastigotes in the supernatant were obtained by centrifugation for 10 min at 124 g. Another Sec22b^+/+^ male mice were left uninfected (control group).

The infected mice were monitored analyzing their general condition, corporal weight and parasitemia. For the latter, a 5 uL sample of tail blood was placed on a slide, covered with a 22 × 22 mm coverslip. Parasitemia was monitored by the Brener method as described in (Brener, 1962).

### 2.3 Spleen and lymph node cell suspensions preparation

At days 6, 10 and 13 post infection (pi), 4 mice from each group were sacrificed and inguinal lymph nodes and spleen were harvested in complete Roswell Park Memorial Institute medium (RPMI, Thermo Fisher Scientific) containing 2 mM L-glutamine, 100 U/mL penicillin, 100 μg/ml streptomycin and 0.1 mM non-essential amino acids and supplemented with 5% Fetal Bovine Serum and mechanically disrupted with a 70 µM-pore filter and syringe plunger, at 4°C. After disruption, spleen suspensions were treated with red blood cell lysis buffer (Gibco) for 3 min at RT. To count and determine viable cells, Trypan Blue 0.4% (Gibco) was used.

### 2.4 Flow cytometry

Initially, the samples were incubated at 4°C for 1 h with Fc receptor binding antibody (Invitrogen) to avoid non-specific binding. Each staining was performed with 100,000 cells. The following antibodies were used: anti-mouse CD3-Brilliant Violet, anti-mouse CD4-FITC, anti-mouse CD8-PerCP Cy5.5, TsKb20 tetramer (H- 2K(b)-PE, NIH Tetramer Facility), anti-mouse B220-APCCy7 and anti-mouse MHCII-APCCy7. For the determination of T lymphocytes, CD3, CD4 and CD8 were used as T cell markers, a DUMP exclusion channel was also used using B22O, MHC II and the cell viability marker LIVE/DEAD^tm^ near IR-fluorescent (Invitrogen). All antibodies were purchased from eBioscience. For the staining, cells were incubated for 40 min at 4°C in the dark. A BD FACSCanto^tm^ II flow cytometer was used, and data were analyzed with FlowJo v10.8.1 software.

### 2.5 Statistical analysis

The statistical analysis was performed with the GraphPad Prism 9 software, differences with *p*-values <0.05 were considered significant. Shapiro-Wilk test of normality was performed to decide the use of parametric or non-parametric tests. The statistical tests used in each case are indicated in the legends of the figures.

## 3 Results

### 3.1 The SNARE protein Sec22b participates in the XPt of antigens from *T. cruzi* involved in the priming of naïve CD8^+^ T cells during parasite infection

**FIGURE 1:**
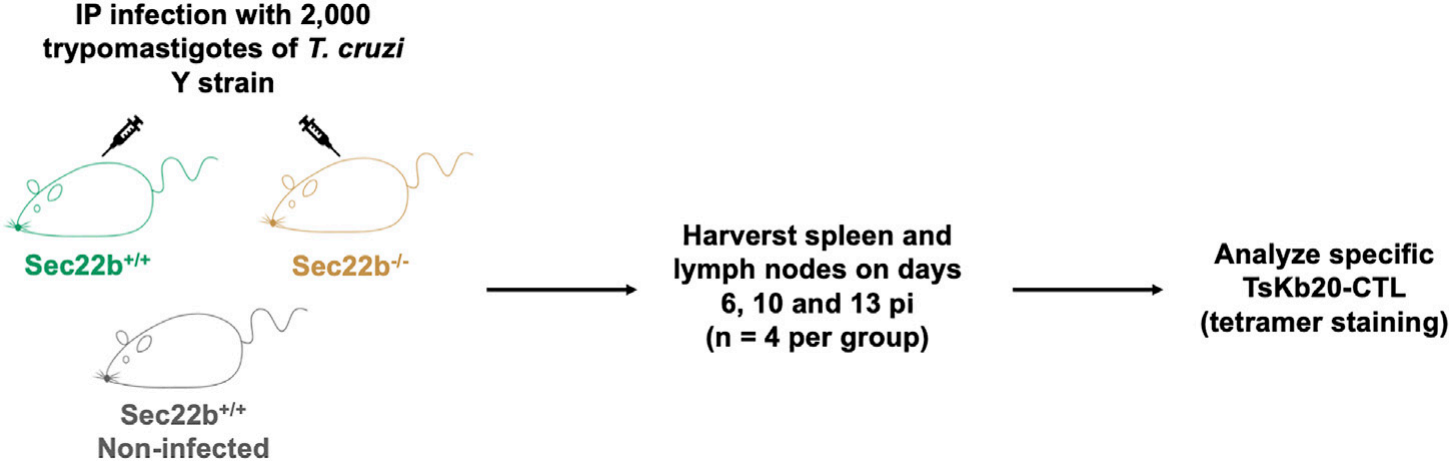
Experimental scheme. Male Sec22b^+/+^ and Sec22b^−/−^ mice were IP infected with 2,000 trypomastigotes of the *T. cruzi* Y strain. As controls, Sec22b^+/+^ mice were left uninfected. At days 6, 10 and 13 post infection four mice per group were euthanized and spleen and lymph nodes were harvested to analyze the specific TsKb20-CD8^+^ T cell response by flow cytometry using tetramer staining.

**FIGURE 2:**
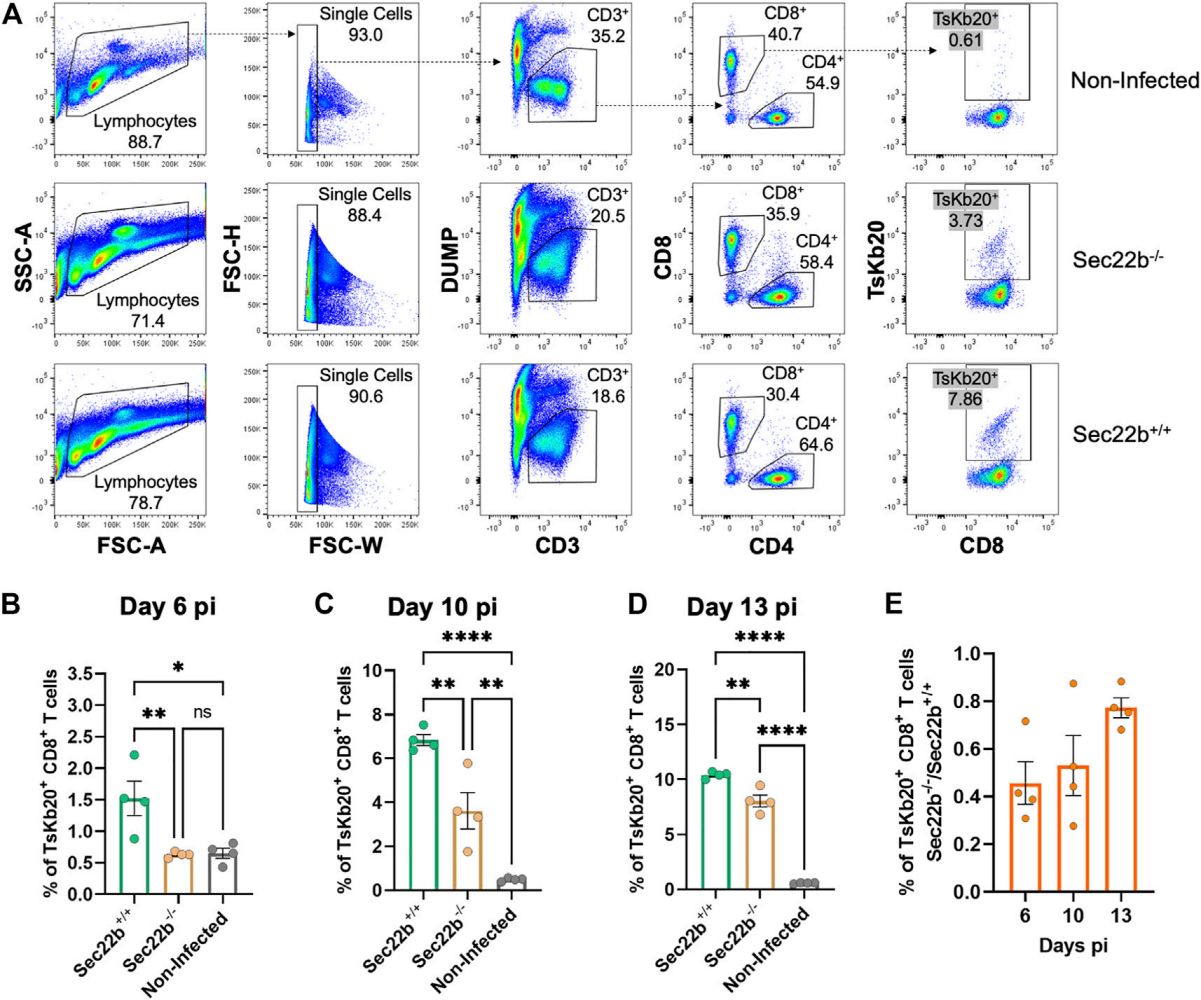
Analysis of the specific TsKb20-CD8^+^ T cells response in spleen. **(A)** Gating strategy used to analyze splenocytes at day 10 pi. After selecting singlets, T lymphocytes were determined as CD3^+^ cells, discriminating from dead cells, B lymphocytes and myeloid cells using a DUMP channel. On the CD3^+^ population, CD8^+^ T cells were selected (as positive cells for the CD8 marker) which were then evaluated with the tetramer to measure specific TsKb20-CD8^+^ T cells. Analysis of the specific TsKb20-CD8^+^ T cells (%) in spleen at day 6 p. i. **(B)** (*p-values*: Sec22b^+/+^ vs. Sec22b^−/−^ 0.0100, Non-Infected vs. Sec22b^+/+^ 0.0115, Non-Infected vs. Sec22b^−/−^ 0.9948); day 10 p. i. **(C)** (*p-values*: Sec22b^+/+^ vs. Sec22b^−/−^ 0.0035, Non-Infected vs. Sec22b^+/+^ <0.0001, Non-Infected vs. Sec22b^−/−^ 0.0042) and day 13 p. i. **(D)** (*p-values*: Sec22b^+/+^ vs. Sec22b^−/−^ 0.0017, Non-Infected vs. Sec22b^+/+^ <0.0001, Non-Infected vs. Sec22b^−/−^ <0.0001). The analysis was performed using One way ANOVA with Tukey’s multiple comparison test, N = 4 **(E)** Ratio of the percentage of specific TsKb20-CD8^+^ T cells found in Sec22b^−/−^ mice over the percentage of TsKb20-CD8^+^ T cells obtained in Sec22b^+/+^ mice. All bar graphs represent the mean with SEM of the parameter analyzed in each case.

To analyze the contribution of Sec22b-dependent XPt to elicit CD8^+^ T cell responses in the context of *T. cruzi* infection, we designed an experimental scheme as summarized in Figure 1. To assess the response of specific effector CD8^+^ T cells, we used a fluorescent multimer composed of four molecules of MHC class I (Kb allele) loaded with the epitope TsKb20 (Rosenberg et al., 2010; Biscari et al., 2022). Figure 2A shows the gating strategy used to determine splenic specific CD8^+^ T cells. Dead cells, B lymphocytes, and myeloid cells were discarded from subsequent analysis with a DUMP channel. Then, on the CD3-positive population, CD4^+^ T lymphocytes were discriminated from CD8^+^ T lymphocytes, and on the latter, TsKb20^+^ cells were quantified using the tetramer. As shown in Figure 2B, at day 6 pi, the population of TsKb20-specific CD8^+^ T cells was significantly higher in the spleen of Sec22b^+/+^ mice compared to Sec22b^−/−^ mice and the control group (uninfected Sec22b^+/+^ mice). No difference was found between specific CD8^+^ T cells in spleens of Sec22b^−/−^ mice and the control group at day 6 pi. At days 10 (Figure 2C) and 13 pi (Figure 2D), XPt-deficient Sec22b^−/−^ mice primed significantly lower TsKb20-CD8^+^ T cells compared with Sec22b^+/+^ littermates.

Interestingly, at day 6 pi Sec22b^−/−^ mice were not able to mount substantial levels of TsKb20-CD8^+^ T cells responses, whereas at days 10 and 13 pi, they raised a significant amount of specific T cells response when compared with uninfected mice (although to a lesser extent than in Sec22b^+/+^ mice, as mentioned). We have previously shown that antigen DPt is fully functional in Sec22b^−/−^ mice (Alloatti et al., 2017). Therefore, the evident increase in the priming of CD8^+^ T cells at days 10 and 13 pi in Sec22b^−/−^ mice is likely originated from the DPt of *T. cruzi* antigens. Remarkably, the contribution of DPt to mount the TsKb20-CD8^+^ T cells response -depicted in Figure 2E as the ratio of TsKb20-CD8^+^ T cells in Sec22b^+/+^ (T cell response primed from both DPt and XPt) and TsKb20-CD8^+^ T lymphocytes in Sec22b^−/−^ (T cell response primed only from DPt)- increases as the infection with *T. cruzi* progresses.

**FIGURE 3:**
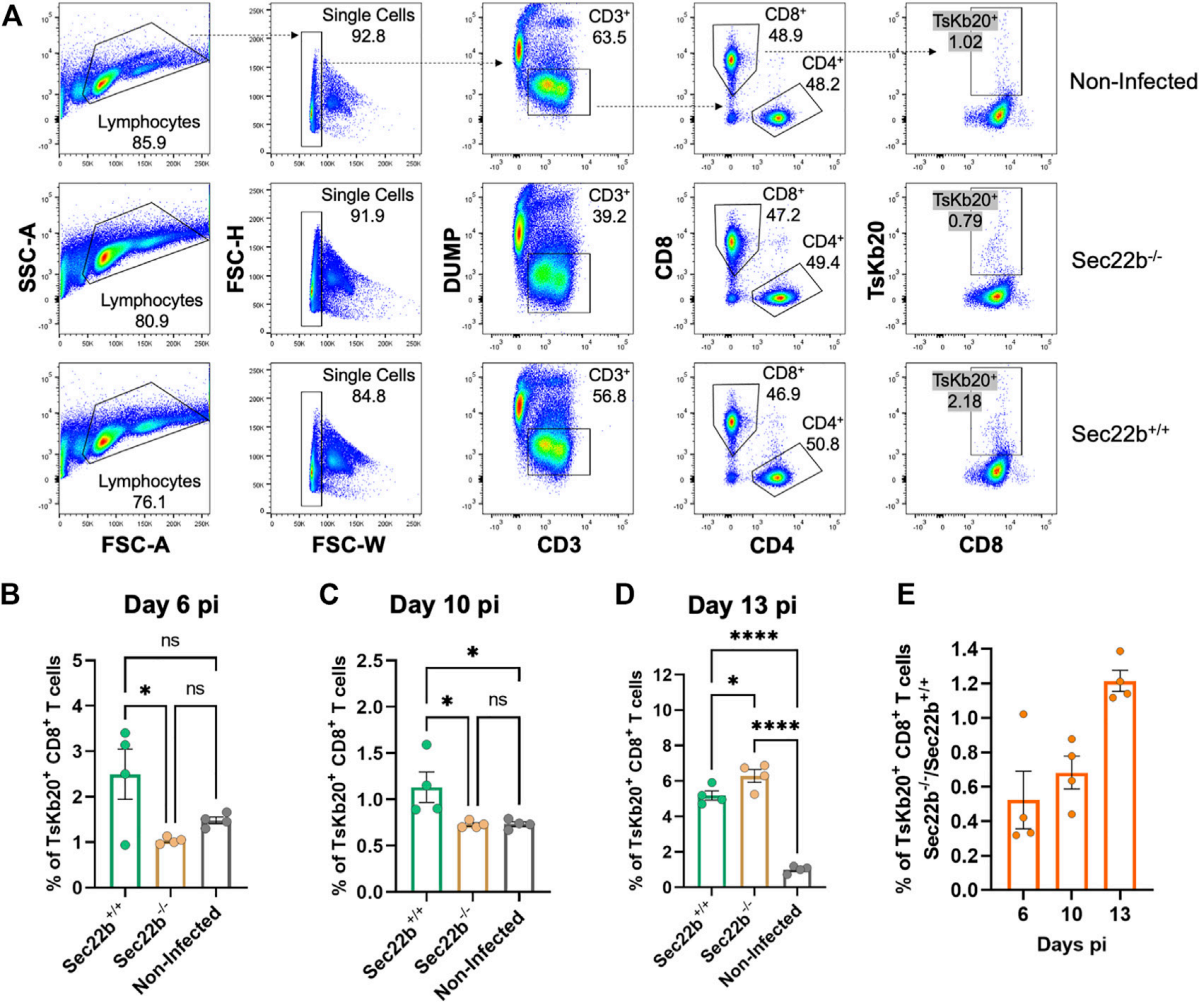
Analysis of the specific TsKb20-CD8^+^ T cells response in lymph nodes. **(A)** Gating strategy used to analyze cells derived from lymph nodes at day 10 pi. As with splenocytes, CD3^+^ cells were selected, excluding dead cells, B lymphocytes, and myeloid cells with a DUMP channel. CD8^+^ T cells were then determined (as positive cells for the CD8 marker) and TsKb20-CD8^+^ T cells were determined as the tetramer positive population. Analysis of the specific TsKb20-CD8^+^ T cells (%) in lymph nodes at day 6 p. i. **(B)** (*p-values*: Sec22b^+/+^ vs. Sec22b^−/−^ 0.0263, Non-Infected vs. Sec22b^+/+^ 0.1194, Non-Infected vs. Sec22b^−/−^ 0.6087; day 10 p. i.) **(C)** (*p-values*: Sec22b^+/+^ vs. Sec22b^−/−^ 0.0377, Non-Infected vs. Sec22b^+/+^ 0.0399, Non-Infected vs. Sec22b^−/−^ 0.9993) and day 13 p. i. **(D)** (*p-values*: Sec22b^+/+^ vs. Sec22b^−/−^ 0.0411, Non-Infected vs. Sec22b^+/+^ <0.0001, Non-Infected vs. Sec22b^−/−^ <0.0001). The analysis was performed using One way ANOVA with Tukey’s multiple comparison test, N = 4 **(E)** Ratio of the percentage of specific TsKb20-CD8^+^ T cells found in Sec22b^−/−^ mice over the percentage of TsKb20-CD8^+^ T cells obtained in Sec22b^+/+^ mice. All bar graphs represent the mean with SEM of the parameter analyzed in each case.

Flow cytometric analysis of inguinal lymph nodes-derived cells is summarized in Figure 3A. The gating strategy was analogous to that used for spleens. The response elicited in lymph nodes of Sec22b^−/−^ mice was significantly impaired at days 6 (Figure 3B) and 10 (Figure 3C) pi compared to Sec22b^+/+^ mice. However, at day 13 pi the contribution of DPt to the priming of the TsKb20-CD8^+^ T cells response completely compensated the deficiency caused by Sec22b silencing (Figure 3D). The growing contribution of DPt to generate specific effector T cells along *T. cruzi* infection is visualized in Figure 3E.

### 3.2 Sec22b-mediated XPt is an important mechanism for immune protection during infection with *T. cruzi*

**FIGURE 4:**
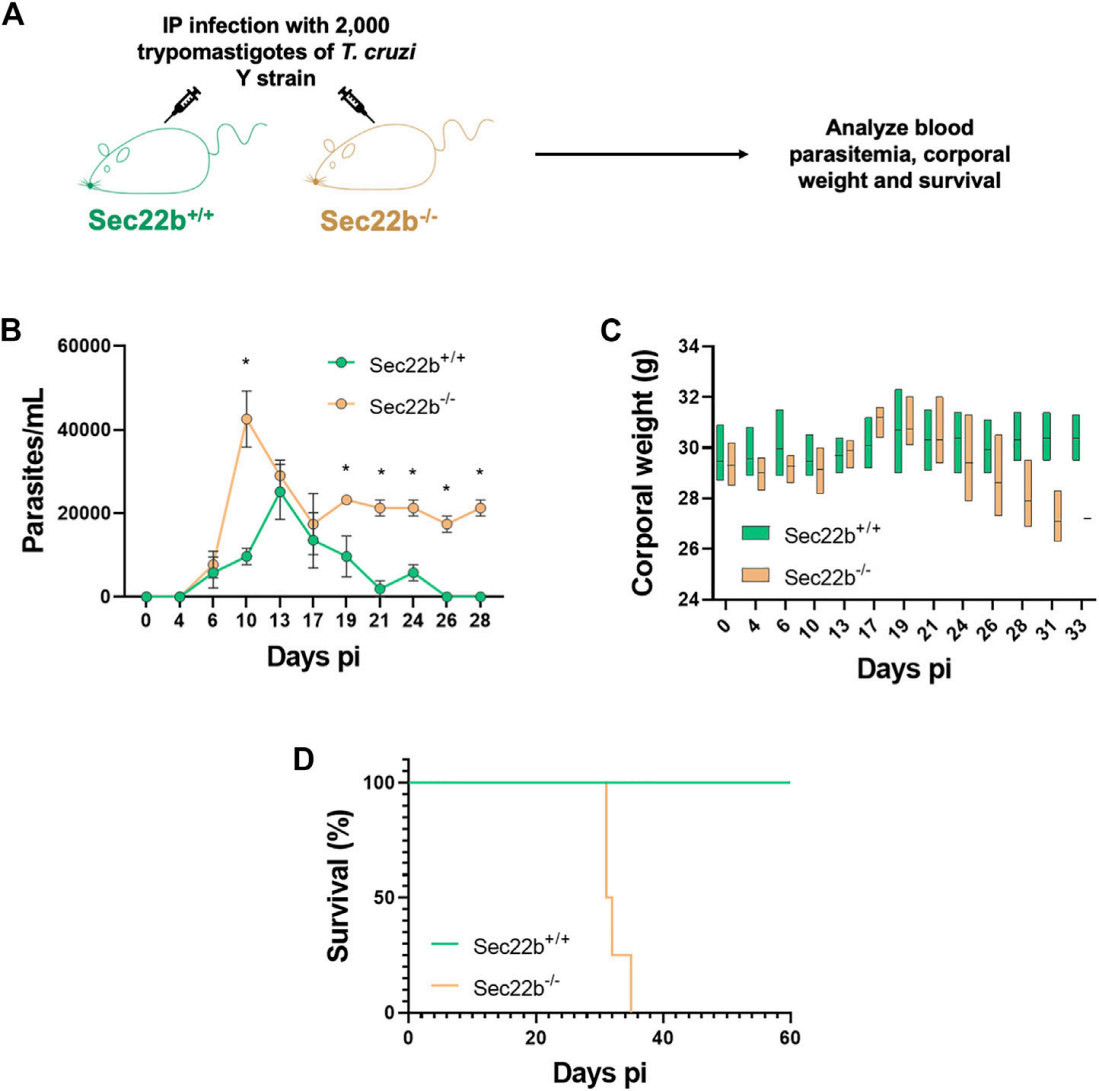
Parasitemia, corporal weight and survival of infected mice. **(A)** Experimental scheme. Sec22b^−/−^ and Sec22b^+/+^ male mice (N = 4 each group) were IP infected with 2,000 *T. cruzi* Y strain trypomastigotes. Blood parasitemia, corporal weight and survival were analyzed over time (up to day 60 pi). **(B)** Parasitemia, expressed as parasites per mL (and represented as mean with SEM), was determined in tail blood, as the number of parasites per ml. Statistical comparison was performed per day using the Mann-Whitney test, all *p-values* < 0.05 at days 10, 19, 21, 24, 26 and 28 dpi (days post infection). **(C)** Corporal weight (g) measured at several days post infection and represented as box plot with line at mean. **(D)** Survival curves for Sec22b^−/−^ and Sec22b^+/+^ mice. Statistical analysis was performed with Log-rank (Mantel-Cox) test, *p-value*: 0.0062.

Next, to study the participation of XPt in the protective capacity of the immune response against *T. cruzi* infection, Sec22b^−/−^ and Sec22b^+/+^ mice were IP infected with 2,000 trypomastigotes of the *T. cruzi* Y strain. Subsequently, infected mice were evaluated over time to monitor parasitemia, corporal weight and survival (Figure 4). As expected, in Sec22b^+/+^ mice the parasite burden reached a maximum in blood on day 13, whereas in Sec22b^−/−^ mice it peaked earlier (at day 10 pi) and remained higher during the experiment (Figure 4A). As shown in Figure 4B, Sec22b^−/−^ mice started losing weight and their condition was deteriorating (not shown) until they finally died, from day 30 pi. Conversely, Sec22b^+/+^ mice, that spontaneously controlled parasitemia, survived during the time studied (up to 60 days). Our model with *T. cruzi* Y strain allows us to establish a sub-lethal infection in C57BL/6 mice when parasites are administered intraperitoneally (Sanoja et al., 2013). The results presented here indicate that XPt in DCs dependent on the Sec22b protein is necessary for the control of *T. cruzi*.

## 4 Discussion

During *T. cruzi* infection CD8^+^ T cells play a critical role in the immune response, as mice depleted of CD8^+^ T cells using antibodies (Tarleton, 1990; Tarleton et al., 1994), as well as mice lacking functional MHC class I molecules (β2-microglobulin-depleted, Tarleton et al., 1992) or CD8 knockout mice (Rottenberg et al., 1993), show higher susceptibility to infection and higher parasite burden. However, even though the CD8^+^ T cell-mediated immunity is vigorous, it is not sufficient to fully eliminate the pathogen from the murine host. These important but deficient CD8^+^ T cell responses are associated with epitope immunodominance (de Alencar et al., 2007; Tzelepis et al., 2008; Rosenberg et al., 2010), delayed kinetics of parasite-specific CTL responses -mainly related to insufficient TLR activation of antigen presenting cells (Tarleton et al., 1992; Padilla et al., 2009a; 2009b), immunosenescence (Argüello et al., 2014) and a potential tolerogenic imprint to infected DCs (Alba Soto et al., 2010; Poncini et al., 2010; 2015). Some similarities have been described in humans, wherein patients with whole-blood transcriptional signatures enriched in genes related to CD8^+^ T cell cytotoxicity show reduced parasitism as well as less severe chagasic chronic symptoms (Laugier et al., 2017; Acosta Rodríguez et al., 2019). Also, during the chronic phase, cardiac severity was associated with exhaustion of CD8^+^ T cells (Albareda et al., 2006). Such results highlight the significance of effector CD8^+^ T cell responses for protection during *T. cruzi* infection.

To date, many MHC class I-restricted epitopes have been described in *T. cruzi*, being those derived from proteins of the trans-sialidases family the most studied, in particular the epitope TsKb20 (Martin et al., 2006; Tzelepis et al., 2008; Ferragut et al., 2021). *T. cruzi*, unlike other parasites, has been shown not to impair—indeed in some cases upregulate—antigen processing and presentation in MHC class I, at least *in vitro* (Buckner et al., 1997). Non-etheless, the contribution of both MHC class I antigen presentation mechanisms (DPt or XPt) to mount CD8^+^ T cell responses were not evaluated before, mainly due to the lack of appropriate tools. As already mentioned, XPt is important in the development of the CD8^+^ T cell response in other parasites related to *T. cruzi* such as *T. gondii* and *L. major* (Bertholet et al., 2006; Mashayekhi et al., 2011; Ashok et al., 2014; Martínez-López et al., 2015).

In this work we used the Sec22b^−/−^ mouse line to assess the impact of XPt during *T. cruzi* infection. We showed that Sec22b-dependent XPt is important for mounting murine CD8^+^ T cell responses (in particular, specific for TsKb20) which, in turn, are essential for controlling infection with the *T. cruzi* Y strain, as evidenced by increased parasitemia, decreased body weight and decreased survival of XPt-impaired (Sec22b^−/−^) mice. This deficient infection control is associated with a clear reduction in the priming of early specific CD8^+^ T cell responses in secondary lymphoid organs, although in lymph nodes the CD8^+^ T cell response finally reaches similar values in both Sec22b^−/−^ and Sec22b^+/+^ mice at day 13 pi. According to the intracellular life cycle of *T. cruzi* (Supplementary Figure S1A), DCs can present different sources of parasite antigens: a) XPt of antigens from internalized trypomastigotes before they reach the cytosol (antigens dumped to the cytosol in step 3 of Supplementary Figure S1A), b) DPt of antigens generated during amastigote or trypomastigote replication, c) XPt of dead parasites or parasite debris after cell lysis, that have been phagocytosed by uninfected DCs, processed and presented to activate CD8^+^ T cells (Supplementary Figure S1B) and d) XPt of antigens obtained by the internalization of infected or apoptotic tissue non-APC with parasitic peptides loaded onto MHC class I molecules (Supplementary Figure S1C). Sec22b possibly orchestrates the XPt of all antigen sources, either in infected or uninfected DCs. Interestingly, we showed that as the infection with *T. cruzi* develops, the priming of *T. cruzi*-specific responses increases even in XPt-deficient mice, likely promoted by the direct presentation of cytosolic antigens generated upon amastigote intracellular replication. Nevertheless, this compensation was not sufficient to control the *T. cruzi* Y strain infection in XPt deficient mice, which would otherwise trigger a sublethal infection (Sanoja et al., 2013). This represents, to our knowledge, the first evidence of the relevance of antigen XPt in the priming of CD8^+^ T cells during *T. cruzi* infection.

According to the results obtained in this report, we consider that the Sec22b^−/−^ mouse line constitutes an appropriate model to study the relevance of XPt in the development of the immune response induced against different pathogens, without affecting other important functions of DCs such as classical MHC class II presentation, DPt or cytokine production (Mashayekhi et al., 2011; Martínez-López et al., 2015). Although two main pathways of XPt have been described—cytosolic and vacuolar (Cruz et al., 2017) –, here we demonstrate that the cytosolic Sec22b dependent-XPt plays a critical role in the activation of CD8^+^ T cells, an important branch of the immune response against the infection caused by *T. cruzi* in mice and humans.

These findings are of relevance for the development of vaccine strategies aimed to induce or boost cytotoxic responses using adjuvants that specifically stimulate XPt. Studies on vaccines that induce CD8^+^ T cell responses have shown to be relevant for the generation of protective immunity against *T. cruzi* infection (Araújo et al., 2005; Miyahira et al., 2005; Hoft et al., 2007; Bustamante et al., 2008; Bontempi et al., 2015; Biscari et al., 2022). Hence, we have shown that in DCs, the SNARE protein Sec22b facilitates the XPt of *T. cruzi* antigens that are necessary for the control of parasite replication in mice. Deficient XPt directly correlates with the impaired priming of specific CD8^+^ T cells.

## Data Availability

The original contributions presented in the study are included in the article/Supplementary Material, further inquiries can be directed to the corresponding author.
