# Supplementary figures and images for: Sec22b-dependent antigen cross-presentation is a significant contributor of T cell priming during infection with the parasite Trypanosoma cruzi
Source: Front Cell Dev Biol. 2023 Mar 1;11:1138571. doi: 10.3389/fcell.2023.1138571 (PMC10014565; doi:10.3389/fcell.2023.1138571)

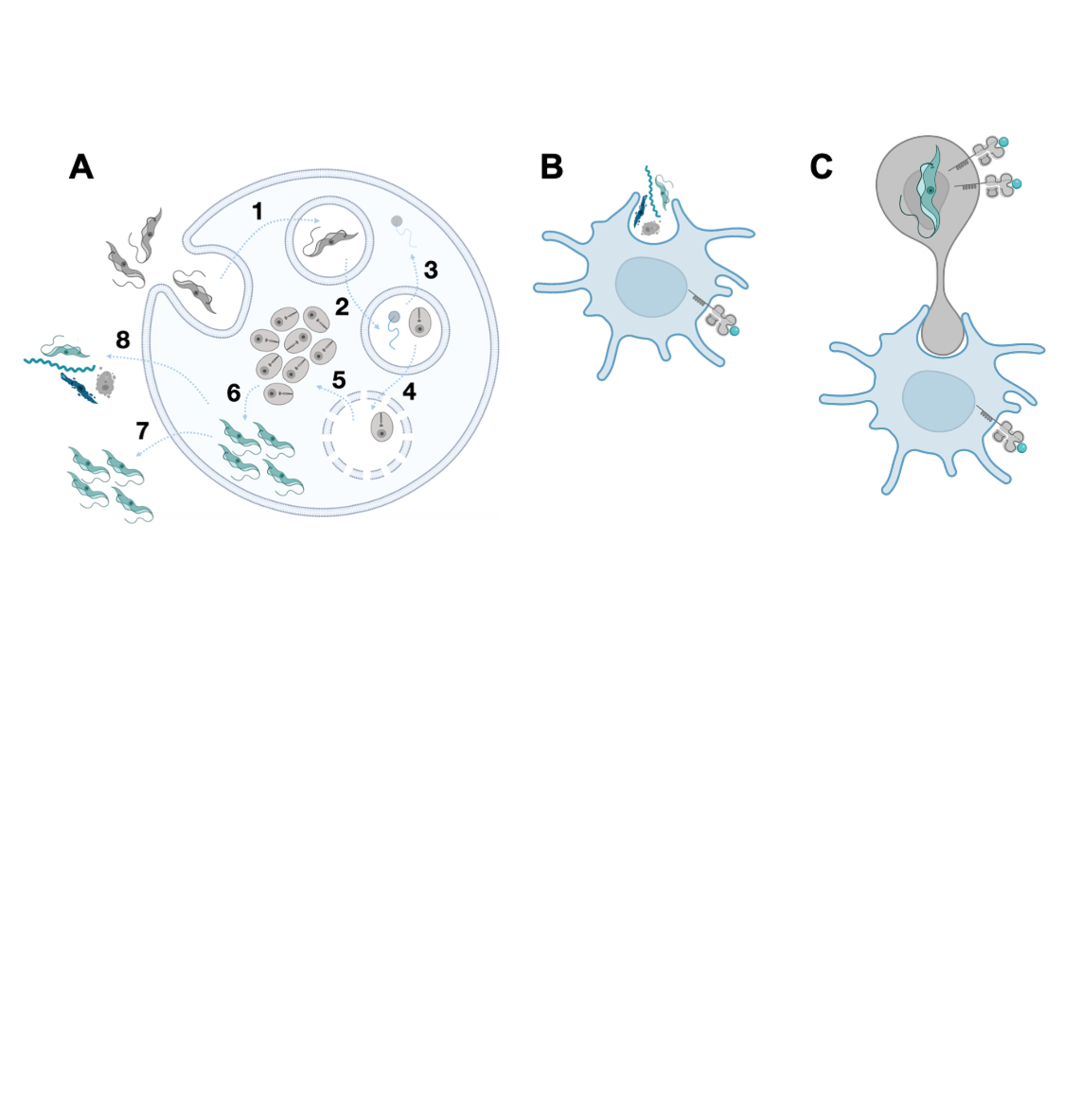

Supplement: Supplementary file 1 [file Image1.TIFF]
